# Suspected Bulbar-Predominant Myasthenia Gravis Exacerbation in an Older Adult With Respiratory Deterioration and Silent Aspiration: A Case Report

**DOI:** 10.7759/cureus.116413

**Published:** 2026-09-17

**Authors:** Hazim I Al Sane, Assim I Al Sane, Riman H Wahidi, Derick G Panackal, Muhammad A Shahzad

**Affiliations:** 1 Medicine, University Hospital Sharjah, Sharjah, ARE; 2 College of Medicine, University of Sharjah, Sharjah, ARE; 3 College of Medicine, Arab American University, Talfit, PSE; 4 School of Medicine, St. George's University, True Blue, GRD; 5 Internal Medicine, University of Chicago, Chicago, USA

**Keywords:** bulbar myasthenia gravis, dysphagia, elderly patient, myasthenia gravis, myasthenic exacerbation, respiratory deterioration, silent aspiration, videofluoroscopic swallow study

## Abstract

Myasthenia gravis (MG) is an autoimmune disorder of the neuromuscular junction characterized by fluctuating skeletal muscle weakness. Bulbar-predominant exacerbations may be difficult to recognize in older adults with multiple comorbidities, particularly when respiratory deterioration is initially attributed to cardiopulmonary disease. We report the case of an 83-year-old woman with generalized acetylcholine receptor antibody-positive MG, dementia with behavioral disturbance, moderate chronic obstructive pulmonary disease (COPD), chronic kidney disease, and previous deep vein thrombosis who was admitted to a psychiatric unit because of worsening paranoia and suspected delusions. During hospitalization, she developed progressive hypoxemia requiring transfer to the medical ward. Initial evaluation considered pulmonary infection, aspiration-related complications, and COPD exacerbation. Neurological examination subsequently demonstrated new predominantly facial and bulbar weakness with preserved limb strength, accompanied by progressive dysphagia and impaired secretion clearance. Videofluoroscopic swallow study demonstrated significant silent aspiration of thin and mildly thickened liquids; however, the etiology of the dysphagia and aspiration remained potentially multifactorial. Serial arterial blood gases demonstrated persistent hypercapnia; however, stable PaCO₂ values, preserved pH, markedly elevated bicarbonate, and positive base excess suggested a substantial chronic hypercapnic component with concomitant metabolic alkalosis, with underlying COPD representing an important potential contributor. In the context of known generalized MG and newly documented facial and bulbar dysfunction, a suspected bulbar-predominant myasthenic exacerbation was considered a potentially important contributor within a multifactorial respiratory deterioration rather than the sole cause. She was treated with intravenous immunoglobulin and escalation of corticosteroid therapy. Although her respiratory status stabilized, persistent bulbar dysfunction, dysphagia, and frailty remained, and following goals-of-care discussions, the family elected hospice care. This case emphasizes the diagnostic complexity of respiratory deterioration in older patients with MG and the importance of recognizing new bulbar dysfunction while carefully considering competing pulmonary, aspiration-related, cardiogenic, and functional contributors.

## Introduction

Myasthenia gravis (MG) is a chronic autoimmune disorder of the neuromuscular junction characterized by fluctuating skeletal muscle weakness and fatigability [1]. Although ocular manifestations are common, involvement of bulbar and respiratory musculature may cause dysphagia, dysarthria, impaired airway protection, ineffective cough, and, in severe cases, respiratory compromise requiring ventilatory support [1,2]. Recognition of these manifestations is particularly important because respiratory deterioration remains a major source of morbidity in MG [2-4].

Bulbar dysfunction may be clinically subtle and can predispose patients to aspiration without overt choking or an effective cough. Instrumental swallowing assessment, including videofluoroscopic swallow study (VFSS), can identify clinically occult swallowing dysfunction and aspiration risk and guide dietary modification and aspiration precautions [5,6]. In older adults, however, recognition of a myasthenic contribution may be complicated by chronic obstructive pulmonary disease (COPD), heart failure, infection, frailty, cognitive impairment, and other potential causes of respiratory deterioration [2-4].

We report the case of an elderly woman with acetylcholine receptor (AChR)-positive generalized MG who developed respiratory deterioration during psychiatric hospitalization. Despite preserved limb strength and absence of prominent ocular findings, neurological evaluation demonstrated new facial and bulbar weakness with progressive dysphagia, while VFSS independently identified significant silent aspiration. Underlying COPD, cognitive impairment, frailty, and competing cardiopulmonary processes complicated attribution of her respiratory decline. This case illustrates how occult bulbar deterioration and silent aspiration may be overlooked when more common pulmonary and functional explanations initially dominate the clinical assessment.

## Case presentation

An 83-year-old woman who was a resident of a nursing facility, with a medical history significant for generalized MG, dementia with behavioral disturbance, moderate COPD, chronic kidney disease stage 3, celiac disease, vitamin D deficiency, breast cancer, and previous deep vein thrombosis (DVT) managed with apixaban, was admitted to the psychiatric unit because of worsening paranoia and suspected delusions (Hospital Day 1). She was a former smoker who had stopped smoking more than 20 years before admission. Her generalized MG had been managed in the outpatient setting with prednisone 2.5 mg twice daily (5 mg/day), and previous AChR antibody testing was positive. Testing for muscle-specific kinase (MuSK) and low-density lipoprotein receptor-related protein 4 (LRP4) antibodies, previous repetitive nerve stimulation (RNS) or single-fiber electromyography (SFEMG), and Myasthenia Gravis Foundation of America (MGFA) clinical classification at diagnosis or before admission were not available in the records reviewed. The duration of prednisone therapy and the rationale for maintenance at this relatively low dose were also not documented. Prior use of pyridostigmine or other immunosuppressive therapies, including reasons for their discontinuation if previously prescribed, could not be established from the available records. There was no documented recent MG exacerbation, diplopia, ptosis, generalized weakness, previous myasthenic crisis, baseline dysphagia, or prior bulbar or respiratory involvement. A history of thymoma or thymectomy was not documented. Although the absence of recently documented MG manifestations suggested no recognized acute deterioration before admission, the available records were insufficient to formally characterize her pre-admission MG as clinically stable.

At baseline, she was ambulatory with a walker and required assistance with activities of daily living, and she did not require supplemental oxygen. Previous pulmonary function testing was not available. Her pre-admission medications included prednisone 2.5 mg twice daily, apixaban (Eliquis) 2.5 mg every 12 hours, budesonide/formoterol (Symbicort) 160/4.5 mcg two puffs twice daily, acetaminophen 500 mg as needed, and benzocaine-menthol lozenges as needed for sore throat.

On Hospital Day 3, the patient developed progressive dyspnea associated with oxygen desaturation and was transferred to the medical ward for further evaluation. She required supplemental oxygen via nasal cannula at 3 L/minute. Pulmonology was consulted because of persistent hypoxemia despite oxygen therapy. Clinical examination revealed significant upper-airway congestion, and her oxygen saturation improved following oral and nasopharyngeal suctioning, suggesting that retained upper-airway secretions contributed to the hypoxemia. Given her subsequent objective bulbar weakness and progressive dysphagia, impaired secretion clearance related to bulbar dysfunction was considered a possible contributing mechanism; however, a direct causal relationship could not be established.

Empiric piperacillin-tazobactam and bronchodilator therapy were initiated while pulmonary infection, aspiration-related complications, and COPD exacerbation remained part of the differential diagnosis. However, there was no substantial improvement in her respiratory status following bronchodilator therapy, and pulmonology considered an acute COPD exacerbation less likely based on the overall clinical presentation. At the time of respiratory deterioration, the patient was afebrile, and chest radiography demonstrated clear lungs without focal consolidation or pleural effusion. Infectious evaluation demonstrated negative procalcitonin, negative respiratory viral polymerase chain reaction (PCR) testing, negative sputum culture, negative urinalysis and urine culture, and blood cultures with no growth. C-reactive protein was mildly elevated at 12.2 mg/L. Taken together, these findings did not provide clear evidence of an acute pulmonary infection, although infection could not be definitively excluded. As respiratory symptoms persisted, neurological evaluation was requested because of her known history of MG and progressive swallowing difficulty.

Neurological examination demonstrated preserved limb strength (5/5 throughout) with objective facial and bulbar weakness, including bilateral orbicularis oculi weakness (4/5), orbicularis oris weakness (4/5), and tongue weakness (4/5). Extraocular movements were intact without ptosis or diplopia, and fatigability could not be reliably reproduced during examination. Additional assessment of neck flexion and extension, palate elevation, speech quality and dysarthria, jaw closure, cough strength, respiratory pattern, deep tendon reflexes, sensory function, and fluctuation of weakness was not consistently documented in the available records and therefore could not be reliably reconstructed retrospectively. The patient reported worsening swallowing difficulty despite denying overt choking episodes. Neurology recommended serial monitoring of negative inspiratory force and vital capacity; however, no reliable numeric measurements could be obtained because of limitations in patient participation, and therefore serial changes in respiratory muscle strength could not be assessed (Figure 1).

**Figure 1:**
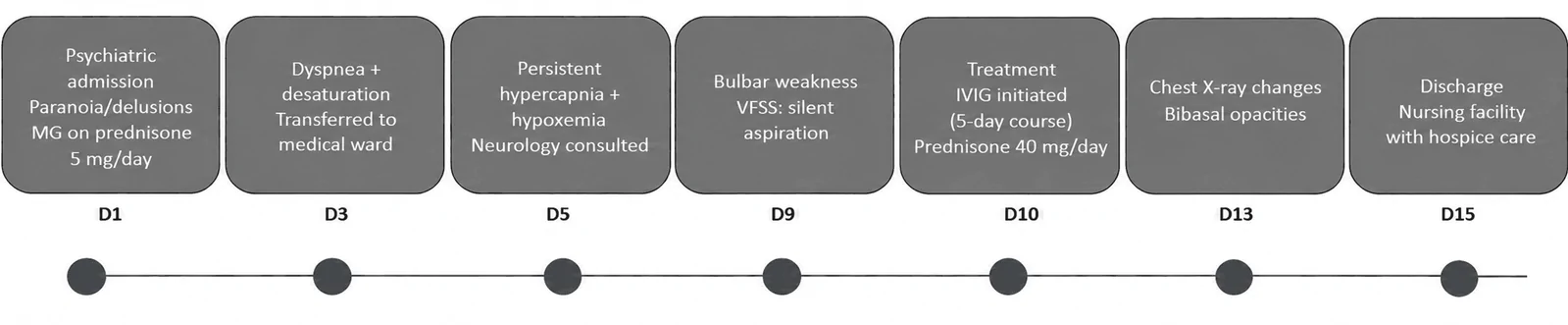
Clinical timeline of the patient's hospital course Timeline showing psychiatric admission, onset of respiratory symptoms, transfer to the medical ward, neurological consultation, videofluoroscopic swallow study, initiation of intravenous immunoglobulin, and discharge D, hospital day The figure was created by the authors using Microsoft PowerPoint (Microsoft Corporation, Redmond, Washington, United States)

Investigations

Chest radiography obtained on Hospital Day 3 demonstrated no acute cardiopulmonary abnormality, with clear lungs and no evidence of focal consolidation, pleural effusion, or pneumothorax. Repeat chest radiography on Hospital Day 5 demonstrated mild-to-moderate bilateral interstitial prominence without pleural effusion or pneumothorax. A subsequent radiograph obtained on Hospital Day 13 demonstrated mild central pulmonary vascular congestion, small-to-moderate left and small right pleural effusions, and bibasal hazy opacities (Figure 2). These findings were nonspecific and, in the context of documented dysphagia and aspiration risk, could reflect aspiration-related pulmonary changes; however, the accompanying pulmonary vascular congestion and pleural effusions also raised the possibility of cardiogenic or volume-related processes. Heart failure or volume overload could not be definitively excluded based on the available evaluation, and superimposed infection also remained possible.

**Figure 2:**
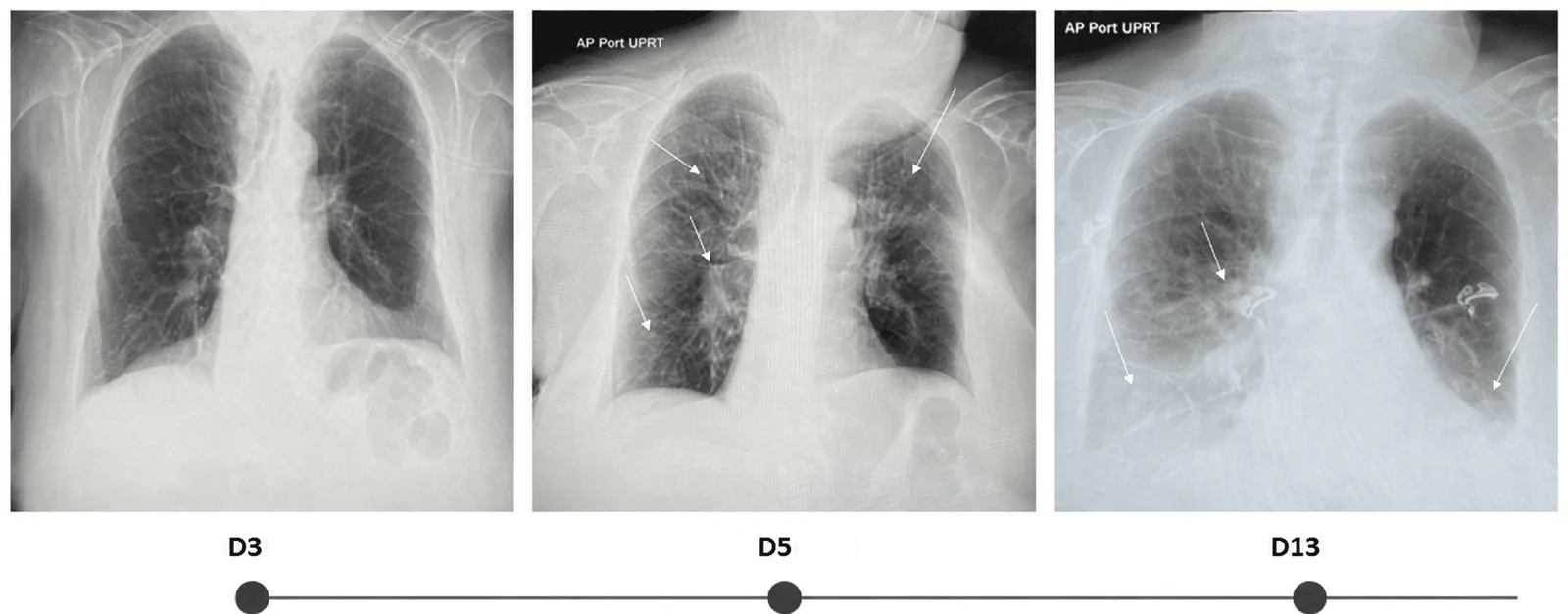
Serial chest radiographs D3: No acute cardiopulmonary abnormality D5: Mild-to-moderate bilateral interstitial prominence without focal consolidation (arrows) D13: Mild central pulmonary vascular congestion with bilateral pleural effusions and bibasal hazy opacities (arrows) D, hospital day

Because of progressive dysphagia and concern for impaired airway protection, a videofluoroscopic swallow study was performed on Hospital Day 9. The study demonstrated premature spillage of thin and mildly thickened liquids with significant silent laryngeal aspiration. Thin liquids administered by straw resulted in silent aspiration with delayed cough. No aspiration was observed with moderately thickened liquids or pudding consistency. The VFSS objectively established swallowing dysfunction and aspiration but did not determine their underlying etiology; given the patient’s advanced age, dementia, frailty, and underlying neuromuscular disease, the dysphagia and aspiration were considered potentially multifactorial. Representative videofluoroscopic images demonstrating silent laryngeal aspiration are shown in Figure 3, and the detailed swallowing findings are summarized in Table 1.

**Figure 3:**
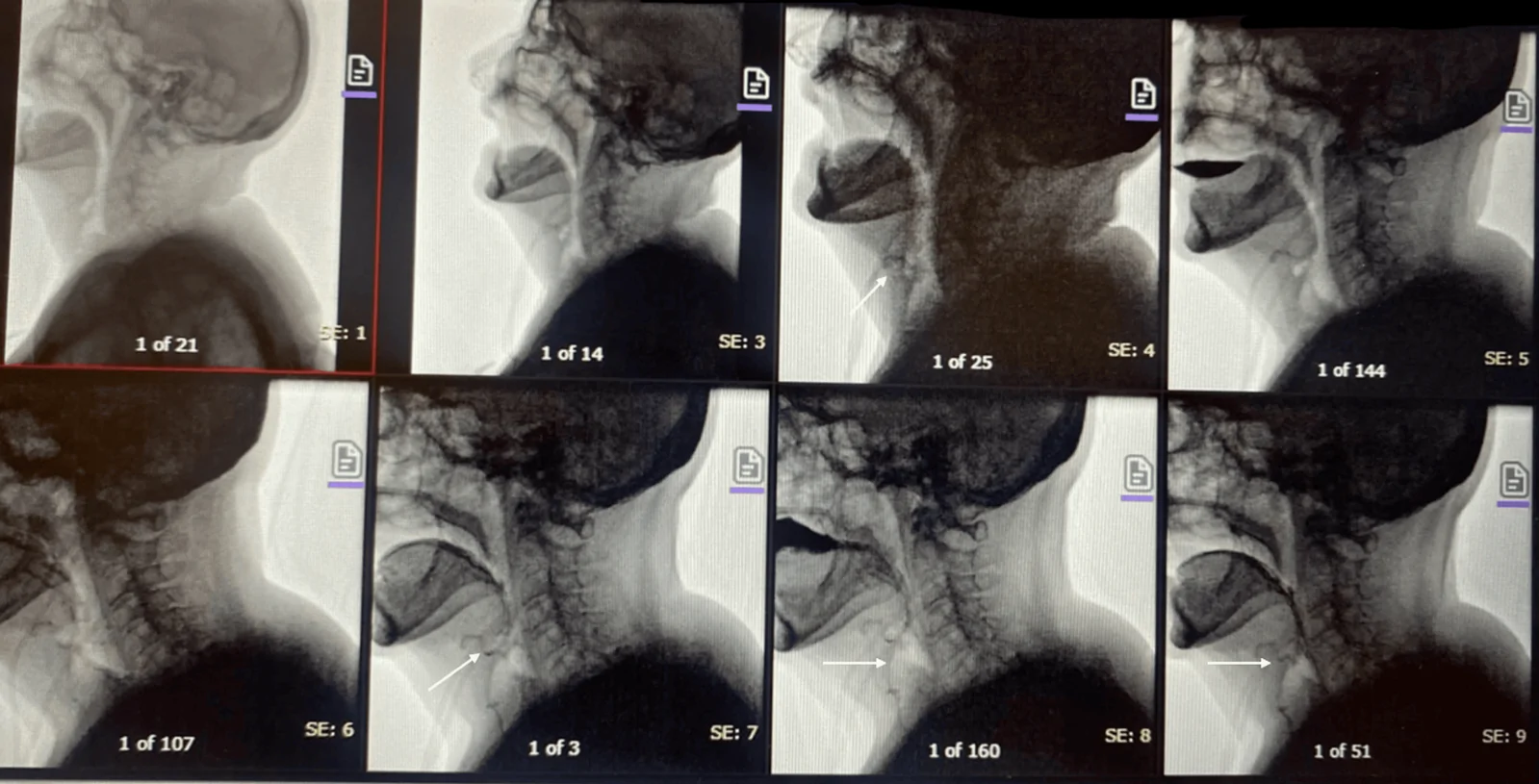
Videofluoroscopic swallow study demonstrating silent laryngeal aspiration Representative videofluoroscopic images demonstrating premature spillage and silent laryngeal aspiration of thin and mildly thickened liquids. Arrows indicate aspirated contrast material. Thin liquids administered by straw also resulted in silent aspiration with a delayed cough. No aspiration was observed with moderately thickened liquids or pudding consistency.

**Table 1:** Videofluoroscopic swallow study findings

Bolus consistency	Findings
Thin liquids	Premature spillage with significant silent laryngeal aspiration
Mildly thickened liquids	Premature spillage with significant silent laryngeal aspiration
Thin liquids (straw)	Silent aspiration with delayed cough
Moderately thickened liquids	No aspiration
Pudding consistency	No aspiration

Serial arterial blood gas analyses demonstrated persistent hypercapnia with associated hypoxemia despite supplemental oxygen therapy (Table 2). Partial pressure of carbon dioxide in arterial blood (PaCO₂) remained essentially unchanged between Hospital Day 5 and Hospital Day 11 (56.8 and 57.0 mmHg, respectively), while pH remained preserved and serum bicarbonate was markedly elevated (37.6 and 39.7 mmol/L, respectively), with corresponding positive base excess values. This pattern was more consistent with a substantial chronic hypercapnic component accompanied by metabolic alkalosis than with progressive acute hypercapnia. Underlying COPD represented an important potential contributor to the chronic hypercapnia. Accordingly, the arterial blood gas findings alone could not establish acute MG-related ventilatory failure.

**Table 2:** Serial arterial blood gas measurements Arrows indicate values above (↑) or below (↓) the reference range. D, hospital day

Hospital Day	FiO_2_%	pH	PaCO_2 _(mmHg)	PaO_2 _(mmHg)	HCO_3_- (mmol/L)	Base Excess (mmol/L)	Oxygen saturation (%)
D5	32	7.39	56.8 ↑	69.8 ↓	37.6 ↑	12.7 ↑	93.8 %
D11	45	7.45	57.0 ↑	71.8 ↓	39.7 ↑	15.7 ​↑	96.1 %

Laboratory investigations demonstrated neutrophilia on admission, with subsequent development of leukocytosis and more pronounced absolute neutrophilia during respiratory deterioration. Procalcitonin was negative, respiratory viral polymerase chain reaction (PCR) testing was negative, and blood cultures showed no growth. The infectious evaluation did not provide clear evidence of an acute pulmonary infection (Table 3).

**Table 3:** Pertinent laboratory investigations Arrows indicate values above (↑) or below (↓) the reference range. An em dash (—) indicates that the investigation was not performed on that hospital day. WBC, white blood cell count; CRP, C-reactive protein; PCR, polymerase chain reaction; D, hospital day.

Investigation	Reference Range	D1 (18 June)	D5 (22 June)
WBC (×10³/µL)	4.0–10.0	9.76	15.29 ↑
Neutrophils (%)	40–75	83.0 ↑	81.1 ↑
Absolute neutrophil count (×10³/µL)	1.5–7.5	8.09 ↑	12.40 ↑
Lymphocytes (%)	20–45	9.9 ↓	6.9 ↓
CRP (mg/L)	<5.0	12.2 ↑	9.3 ↑
Procalcitonin	Negative	Negative	—
Respiratory viral PCR	Negative	Negative	—
Blood cultures	No growth	No growth	—
Sputum culture	Negative	Negative	—
Urinalysis	Negative	Negative	—
Urine culture	Negative	Negative	—

Differential diagnosis

The initial differential diagnosis included pneumonia, aspiration pneumonitis, COPD exacerbation, and acute decompensated heart failure. Pulmonary infection was initially considered given the leukocytosis, neutrophilia, and mildly elevated CRP; however, it became less strongly supported in the absence of fever, with negative procalcitonin, blood cultures, respiratory viral testing, and sputum culture, together with no focal consolidation on early chest imaging. Nevertheless, pulmonary infection, including aspiration-related infection, could not be definitively excluded. An acute COPD exacerbation was also considered given her underlying moderate COPD; however, the absence of substantial improvement in respiratory status following bronchodilator therapy and the overall clinical assessment made an acute exacerbation less likely as the primary explanation for her deterioration. Underlying COPD nevertheless remained an important potential contributor to her chronic hypercapnia.

VFSS independently demonstrated silent aspiration, which was considered an additional potential contributor to respiratory compromise but did not establish a myasthenic etiology for the dysphagia. Possible cardiogenic or volume-related contributions were also considered, particularly after later imaging demonstrated pulmonary vascular congestion and bilateral pleural effusions, and could not be definitively excluded. Given the patient’s advanced age, dementia, frailty, and multiple comorbidities, nonspecific functional decline and deconditioning were additional potential contributors. Medication-related effects were reviewed; however, potentially respiratory-depressing medications ordered as needed were not administered. Thus, the objective findings established new facial and bulbar weakness in a patient with known AChR-positive MG, hypoxemia requiring supplemental oxygen, persistent hypercapnia with a substantial chronic component, and VFSS-confirmed silent aspiration. These findings did not establish a single unifying etiology. The interpretation of a bulbar-predominant myasthenic exacerbation was therefore a clinical inference based principally on the new neurological findings and was considered one potential contributor within a multifactorial deterioration that also included underlying COPD, aspiration-related complications, possible cardiogenic or volume-related disease, dementia, frailty, and deconditioning.

Treatment

The patient received multidisciplinary care involving pulmonology, neurology, speech-language pathology, and internal medicine. Initial management included supplemental oxygen, oral and nasopharyngeal suctioning, bronchodilator therapy, aspiration precautions, dietary modification based on speech-language pathology recommendations, empiric intravenous piperacillin-tazobactam, and continuation of apixaban for her history of DVT. Piperacillin-tazobactam was subsequently discontinued after the infectious evaluation did not provide clear evidence of an acute bacterial pulmonary infection. Her supplemental oxygen requirement improved during the subsequent hospital course. The frequency of airway suctioning was not consistently documented, precluding assessment of whether suctioning requirements decreased over time. Pyridostigmine was not administered during the hospitalization.

Following neurological assessment, immunomodulatory therapy with intravenous immunoglobulin (0.4 g/kg/day for five consecutive days) was initiated. Her chronic corticosteroid regimen was escalated from prednisone 5 mg daily to prednisone 40 mg daily. No standardized or quantitative post-treatment neurological or bulbar assessments and no reliable serial respiratory-muscle measurements were available. Therefore, subsequent improvement in supplemental oxygen requirement could not be attributed specifically to intravenous immunoglobulin (IVIG) or corticosteroid therapy and was not considered confirmatory evidence of MG causality.

Outcome and follow-up

The patient completed a five-day course of IVIG together with high-dose corticosteroid therapy. Despite treatment, she remained significantly debilitated with persistent bulbar dysfunction and dysphagia requiring continued aspiration precautions.

Although her respiratory status stabilized, persistent bulbar dysfunction and dysphagia, frailty, underlying dementia, limited functional reserve, and the overall prognosis were discussed with the family during multidisciplinary goals-of-care conversations. In this context, the family elected to pursue hospice care, and she was discharged to her nursing facility with hospice services on Hospital Day 15.

## Discussion

Myasthenic exacerbations presenting predominantly with bulbar and respiratory manifestations can be difficult to recognize in older adults, particularly when multiple comorbidities provide plausible alternative explanations for deterioration [4,7]. In the current patient, the initial presentation occurred during a psychiatric admission, and the subsequent respiratory decline was appropriately evaluated first for common pulmonary and cardiopulmonary causes. Recognition of a neuromuscular contribution was further complicated by preserved limb strength, absence of ptosis or diplopia, and inability to reliably reproduce fatigability on examination. Bulbar-predominant presentations of MG have previously been described in elderly patients, in whom dysphagia or dysarthria may predominate despite minimal ocular or limb involvement [8,9]. In our patient, the development of new objective facial and bulbar weakness, progressive dysphagia, and impaired secretion clearance in the setting of known AChR-positive generalized MG was considered clinically suggestive of a bulbar-predominant myasthenic exacerbation that may have contributed to the deterioration, while the presence of multiple competing processes prevented attribution of the respiratory course to MG alone.

Myasthenic exacerbation refers to clinically significant worsening of pre-existing MG symptoms that may require escalation of therapy, whereas myasthenic crisis represents the severe end of this spectrum, with respiratory or bulbar weakness requiring invasive or noninvasive ventilatory support [3,7]. We therefore describe the episode as a suspected bulbar-predominant myasthenic exacerbation rather than a myasthenic crisis. Although the patient developed significant respiratory deterioration with hypoxemia, persistent hypercapnia, objective bulbar weakness, and progressive dysphagia, she did not require invasive or noninvasive ventilatory support, and reliable negative inspiratory force and vital capacity measurements could not be obtained. The available evidence was therefore insufficient to classify the episode definitively as a myasthenic crisis. Respiratory compromise in MG may result from weakness of respiratory musculature, reduced cough effectiveness, and impaired management of airway secretions [4,7]. In this patient, oxygen saturation improved following oral and nasopharyngeal suctioning, indicating that retained upper-airway secretions contributed to the hypoxemia. The subsequent finding of bulbar weakness and progressive dysphagia raised the possibility that impaired secretion clearance was partly related to neuromuscular dysfunction, but a direct causal relationship could not be established.

Distinguishing neuromuscular respiratory dysfunction from pulmonary and cardiopulmonary disease was particularly important in this case. Serial arterial blood gases demonstrated essentially unchanged PaCO₂ values of 56.8 and 57.0 mmHg, with preserved pH, markedly elevated bicarbonate levels, and positive base excess. In the absence of a baseline arterial blood gas, this pattern suggested a substantial chronic hypercapnic component with concomitant metabolic alkalosis rather than progressive acute CO₂ retention, with underlying COPD representing an important alternative explanation. The arterial blood gas findings alone therefore could not establish acute MG-related ventilatory failure. An acute COPD exacerbation was considered because of her known moderate COPD; however, there was no substantial improvement in respiratory status following bronchodilator therapy, and pulmonology considered an acute exacerbation less likely based on the overall clinical presentation. Underlying COPD nevertheless remained an important potential contributor to chronic hypercapnia. Pulmonary infection was also initially considered in the setting of leukocytosis, neutrophilia, and mildly elevated CRP. However, the absence of fever, negative procalcitonin, negative respiratory viral testing, negative sputum and blood cultures, and absence of focal consolidation on early chest imaging made acute pulmonary infection less strongly supported, although bacterial or aspiration-related infection could not be definitively excluded. Later pulmonary vascular congestion, bilateral pleural effusions, and bibasal hazy opacities further broadened the differential to include cardiogenic or volume-related processes. Heart failure or volume overload was not definitively excluded, and aspiration pneumonitis and atelectatic changes were also considered possible contributors. These findings support a multifactorial interpretation of the respiratory deterioration rather than a purely neuromuscular process.

Management of significant MG exacerbations may include intravenous immunoglobulin or plasma exchange, with treatment individualized according to clinical severity, MG phenotype, comorbidities, and response [3,7,10]. In this case, neurological assessment prompted treatment with intravenous immunoglobulin at 0.4 g/kg/day for five consecutive days and escalation of prednisone from 5 mg/day to 40 mg/day. Pyridostigmine was not administered during the hospitalization. Her supplemental oxygen requirement subsequently improved, although objective serial measures specifically quantifying the neuromuscular response to IVIG were unavailable, and a causal relationship between treatment and respiratory improvement therefore cannot be established. Persistent bulbar dysfunction and dysphagia remained clinically important despite treatment.

Medication exposure was reviewed because some drugs can worsen neuromuscular or respiratory function in MG [11]. Hydrocodone-acetaminophen 5/325 mg every four hours as needed, hydromorphone 0.5 mg intravenously every 15 minutes as needed, and haloperidol 2 mg intramuscularly every four hours as needed were ordered but not administered, making medication-induced respiratory depression unlikely.

Occult aspiration represented another important component of the clinical picture. Bulbar dysfunction in MG may impair pharyngeal and laryngeal function and increase aspiration risk even in the absence of overt choking or coughing [4,5]. In this patient, bedside symptoms underestimated the severity of swallowing impairment. VFSS demonstrated premature spillage and significant silent laryngeal aspiration with thin and mildly thickened liquids, including aspiration of thin liquids administered by straw with only a delayed cough. Instrumental swallowing assessment has been shown to identify clinically inapparent swallowing abnormalities and aspiration risk in patients with MG [5,6]. Importantly, however, VFSS established the presence and severity of swallowing dysfunction and aspiration; it did not establish that the dysphagia was caused exclusively by MG. Advanced age, dementia, frailty, and overall functional decline represented additional potential contributors. The VFSS nevertheless had immediate clinical value by guiding dietary modification and aspiration precautions. Aspiration pneumonia and aspiration pneumonitis may have variable clinical and radiographic manifestations, making them difficult to distinguish from other causes of respiratory deterioration [12]. Furthermore, pharyngeal residue has been associated with increased aspiration risk on subsequent swallows [13], supporting the importance of objective swallowing evaluation and appropriate risk-reduction strategies in patients with neurogenic dysphagia [14].

The clinical course also illustrates the particular challenges of managing suspected MG exacerbation in a frail elderly patient. Even when a neuromuscular contribution is clinically supported, respiratory deterioration may result from several interacting processes, including impaired secretion clearance, aspiration, chronic pulmonary disease, cardiogenic or volume-related abnormalities, deconditioning, and limited functional reserve. In this patient, respiratory status ultimately stabilized, but persistent bulbar dysfunction and dysphagia, frailty, underlying dementia, limited functional reserve, and overall prognosis remained important considerations. Following multidisciplinary goals-of-care discussions, the family elected hospice care. Thus, management required not only treatment of the suspected MG exacerbation but also aspiration-risk reduction, respiratory support, assessment of competing etiologies, and consideration of the patient's overall functional status and goals of care.

This case has several limitations. Although objective facial and bulbar weakness was documented, fatigability could not be reliably reproduced during examination, reducing the clinical specificity of these findings for an acute MG exacerbation. Reliable bedside measures of respiratory muscle strength, including vital capacity and negative inspiratory force, could not be obtained because of limitations in patient participation, restricting objective assessment of neuromuscular respiratory weakness. Contemporaneous neuroimaging specifically evaluating for a brainstem structural lesion was not available in the records reviewed; therefore, a central neurological contributor to the new bulbar findings could not be definitively excluded. Contemporaneous electrodiagnostic testing and standardized MG severity assessments were also unavailable. Baseline arterial blood gas measurements and previous pulmonary function testing were not available, limiting assessment of the chronicity of the hypercapnia; underlying COPD therefore remained an important alternative contributor. Later pulmonary vascular congestion and pleural effusions raised the possibility of cardiogenic or volume-related contributions that could not be definitively excluded. Dysphagia and aspiration were also potentially multifactorial given the patient's advanced age, dementia, frailty, and underlying neuromuscular disease and therefore could not be attributed exclusively to MG. Finally, standardized quantitative post-treatment assessments of bulbar or neurological function and objective serial measures quantifying response to IVIG were unavailable. These limitations preclude definitive attribution of the respiratory deterioration to MG and support framing the episode as a suspected bulbar-predominant myasthenic exacerbation that may have contributed to a multifactorial clinical deterioration.

## Conclusions

This case highlights the diagnostic challenges of recognizing a possible bulbar-predominant myasthenic exacerbation in elderly patients with multiple comorbidities and respiratory deterioration. In patients with known MG who develop new dysphagia, impaired secretion clearance, or otherwise incompletely explained respiratory worsening, bulbar involvement should remain a diagnostic consideration even when ptosis and diplopia are absent and limb strength is preserved. VFSS can identify clinically occult aspiration and characterize swallowing dysfunction, thereby guiding dietary modification and aspiration-risk reduction, but it does not establish that dysphagia is caused by MG. In medically complex elderly patients, respiratory deterioration may be multifactorial, requiring consideration of neuromuscular dysfunction alongside chronic pulmonary disease, aspiration-related complications, cardiogenic or volume-related processes, medication exposure, frailty, and functional decline.
